# Comparison of thoracolaparoscopic esophagectomy with cervical anastomosis with McKeown esophagectomy for middle esophageal cancer

**DOI:** 10.1186/s12957-015-0727-y

**Published:** 2015-11-05

**Authors:** Hai-Tao Huang, Fei Wang, Liang Shen, Chun-Qiu Xia, Chen-Xi Lu, Chong-Jun Zhong

**Affiliations:** Department of Thoracic and Cardiovascular Surgery, Nantong First People’s Hospital, the Second Affiliated Hospital of Nantong University, No.6 North Hai’er Xiang Road, Nantong, 226001 People’s Republic of China

**Keywords:** Esophageal cancer, Mckeown esophagectomy, Thoracolaparoscopic esophagectomy, Cervical anastomosis

## Abstract

**Background:**

In China, the middle esophageal squamous cell cancer is the most common tumor type, and Mckeown esophagectomy (ME) is preferably adopted by thoracic surgeon. But, the surgical trauma of ME is great. Thoracolaparoscopic esophagectomy (TE) was developed to decrease the operative stress; however, the safety and efficacy were not defined. In this study, clinical outcomes were compared between patients who received ME and TE.

**Methods:**

The data of 113 patients who suffered from middle-thoracic esophageal cancer during the same period were collected. Sixty-two patients received ME (ME group), and 51 patients received TE (TE group). Patients’ demographics and short-term clinicopathologic outcomes were comparable between the two groups. Survival rate was estimated using the Kaplan–Meier method, and comparisons between groups were performed with log–rank test.

**Results:**

Patients in TE group had lower body mass index (BMI). Preoperative tumor stage in TE group was much earlier. Both overall and thoracic operation time were longer in TE group. The blood loss during operation and postoperative day (POD) 1 was less in TE group, which contributed to the less blood transfusion. In TE group, postoperative incidence of pulmonary complications and atrial fibrillation (*p* = 0.035 and *p* = 0.033) was lower; the inflammatory response and incision pain were significantly alleviated; the ICU and in-hospital stay was shorter as well because of less surgical trauma. No statistically significant difference was found between two groups in terms of overall survival or disease-free survival.

**Conclusions:**

The efficacy and safety of TE were supported by the selected patients in this cohort study. Although it is lack of randomness in this research, some advantages of TE were gratifying such as lower postoperative complications and similar survival with ME. A multicenter prospective randomized study is now required.

## Background

Compared with developed countries, in China, the squamous carcinoma in the middle of esophagus as the predominant esophageal cancer (EC) type has led many groups to advocate the thoracolaparoscopic surgical approach with intra-thoracic or cervical anastomosis as the operation of choice for these cancers [1, 2]. Before application thoracolaparoscopic surgical approach, McKeown and Ivor Lewis approaches were used by our department. Compared with left transthoracic approach (one incision in left thorax), combined transabdominal and transthoracic approach was regarded as the optimal strategy for cancer clearance and two-field lymphadenectomy [3–5].

The McKeown approach is thought with numerous advantages over other approaches for middle EC. Potential advantages of the McKeown approach compared to the Ivor Lewis include less chance of local recurrence, anastomosis in neck easier to manage if leak occurs, and less need to expand the thoracic incision since the anastomosis is in the neck instead of the chest [6]. Despite advances in surgical techniques, anesthetic techniques, and perioperative care, morbidity and mortality rates of McKeown or Ivor Lewis approach are consistently high. The most important cause of significant morbidity and mortality after open approaches for EC is the development of cardiac [7, 8] and pulmonary complications [9–11].

In order to reduce the surgical trauma and the postoperative cardiopulmonary complication, minimal invasive thoracolaparoscopic esophagectomy with cervical gastroesophageal anastomosis was performed in our department from 2010. In this study, we reported the clinical outcomes of 51 patients undergoing thoracolaparoscopic esophagectomy with cervical gastroesophageal anastomosis compared with 62 patients undergoing McKeown esophagectomy at the same period. The efficacy and safety of thoracolaparoscopic esophagectomy (TE) would be testified by this study, which could offer theoretical foundation for its clinical application.

## Methods

**Fig. 1:**
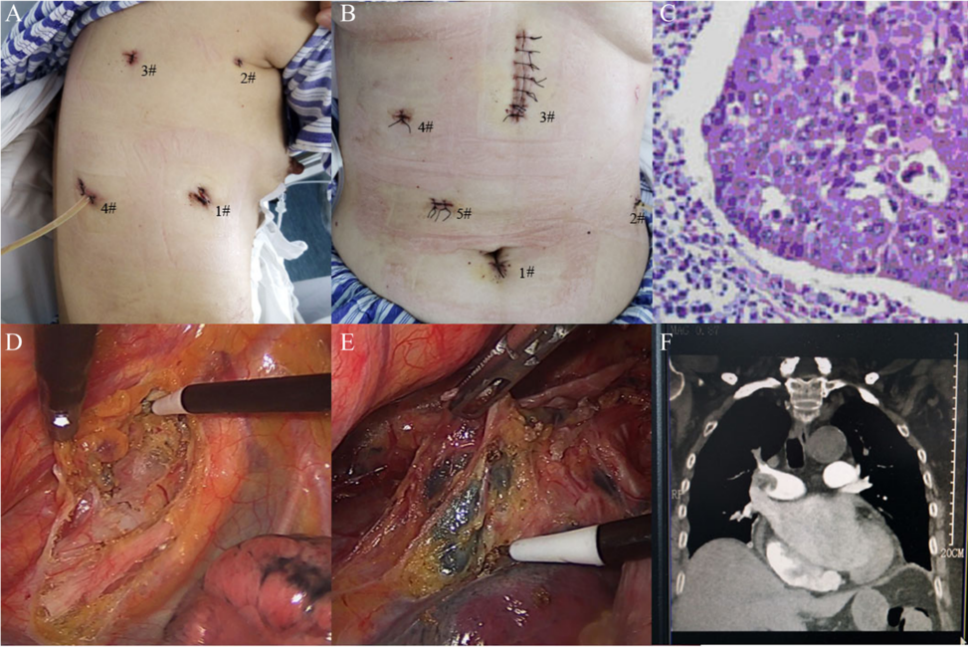
**a** The incisions were made on the right later chest; a negative drainage ball was placed through the 4# port. **b** The incisions were made on abdomen; a negative drainage ball was placed through the 2# port. **c** The histo-pathological type of EC was squamous cell cancer. **d** The lymph nodes along the RLN were dissected. **e** The subcarinal lymph nodes were dissected. **f** One patient suffered pulmonary thrombosis because of DVT, and died in-hospital; in the CT scan, a large embolus was found in the right pulmonary artery trunk

Patients and grouping: from 2010 to 2013, retrospective data of 113 patients with middle esophageal squamous cancer underwent conventional Mckeown esophagectomy (ME) (*n* = 62) or TE (*n* = 51) at Nantong First People’s Hospital were collected. All patients took routine examinations such as blood routine test, blood gas analysis, electrocardiogram, C-response protein, barium swallow, endoscopic ultrasonography for esophagus and stomach with biopsy, and chest and abdominal computed tomography. All patients were discussed at a multidisciplinary specialist team meeting. This study was approved by the ethics committee in Nantong first people’s hospital. The informed consent was obtained from each patient. Then, the standard staging was performed according to local protocols. There was no significant difference in the prevalence of tumor stages in between two groups (showed in Table 1). The histo-pathological types of all patients were squamous cell cancer (Fig. 1c). All operations were mainly performed by surgeon Zhong CJ. Patients with lower BMI and earlier preoperative tumor stage were preferably selected for TE at the beginning of this technique. ME was recommended for patients with higher BMI and advanced tumor stage. No patient received adjuvant therapy before surgery.

**Table 1:** Pre- and postoperative pathological characters

	ME group (*n* = 62)	TE group (*n* = 51)	*p*
Preoperative T stage			<0.0001^#^
Tis	0 (0 %)	3 (5.9 %)	
T1	5 (8.1 %)	15 (29.4 %)	
T2	17 (27.4 %)	25 (49.0 %)	
T3	38 (61.3 %)	8 (15.7 %)	
T4	2 (3.2 %)	0 (0 %)	
Preoperative N stage			
N0	18 (29.0 %)	31 (60.8 %)	0.001*
N1	28 (45.2 %)	16 (31.4 %)	
N2	11 (17.7 %)	4 (7.8 %)	
N3	5 (8.1 %)	0 (0 %)	
Preoperative M stage			
M0	61 (98.4 %)	51 (100 %)	0.362
M1	1 (1.6 %)	0 (0 %)	
Preoperative TNM stage			0.322
IA	5 (8.1 %)	9 (17.6 %)	
IB	11 (17.7 %)	10 (19.6 %)	
IIA	12 (19.4 %)	10 (19.6 %)	
IIB	8 (12.9 %)	11 (21.6 %)	
IIIA	16 (25.8 %)	8 (15.7 %)	
IIIB	7 (11.3 %)	3 (5.9 %)	
IIIC	2 (3.2 %)	0 (0 %)	
IV	1 (1.6 %)	0 (0 %)	
Postoperative T stage			0.001*
Tis	0 (0 %)	2 (3.9 %)	
T1	11 (17.7 %)	11 (21.6 %)	
T2	13 (21.0 %)	27 (52.9 %)	
T3	37 (59.7 %)	11 (21.6 %)	
T4	1 (1.6 %)	0 (0 %)	
Postoperative N stage			0.270
N0	31 (50.0 %)	34 (66.7 %)	
N1	16 (25.8 %)	11 (21.6 %)	
N2	13 (21.0 %)	5 (9.8 %)	
N3	2 (3.2 %)	1 (2.0 %)	
Postoperative M Stage			0.196
M0	60 (96.8 %)	51 (100 %)	
M1	2 (3.2 %)	0 (0 %)	
Number of removed LN	15.3 ± 3.2	17.8 ± 3.6	0.0002*
Vessel cancer embolus	15 (24.2 %)	14 (27.5 %)	0.693
Nerve invasion	11 (17.7 %)	8 (15.7 %)	0.771
Median mD (mm)	31.3 ± 4.2	28.8 ± 3.4	0.001*
Morphology			0.711
Concealed	0 (0 %)	1 (2.0 %)	
Erosive	1 (1.6 %)	2 (3.9 %)	
Plaque	0 (0 %)	1 (2.0 %)	
Polypoid	1 (1.6 %)	0 (0 %)	
Medullary	26 (41.9 %)	18 (35.3 %)	
Fungating	9 (14.5 %)	6 (11.8 %)	
Ulcer	22 (35.5 %)	21 (41.2 %)	
Constrictive	3 (4.8 %)	2 (3.9 %)	
Differentiation			0.626
G1	6 (9.7 %)	4 (7.8 %)	
G2	25 (40.3 %)	27 (52.9 %)	
G3	28 (45.2 %)	18 (35.3 %)	
G4	2 (3.2 %)	2 (3.9 %)	
Gx	1 (1.6 %)	0 (0 %)	
Postoperative TNM stage			0.197
IA	6 (9.7 %)	8 (15.7 %)	
IB	5 (8.1 %)	9 (17.6 %)	
IIA	7 (11.3 %)	10 (19.6 %)	
IIB	12 (19.4 %)	11 (21.6 %)	
IIIA	17 (27.4 %)	7 (13.7 %)	
IIIB	10 (16.1 %)	5 (9.8 %)	
IIIC	3 (4.8 %)	1 (2.0 %)	
IV	2 (3.2 %)	0 (0 %)	
Postoperative chemotherapy	42 (67.7 %)	28 (54.9 %)	0.162
Postoperative chemotherapy + radiotherapy	19 (30.6 %)	11 (21.6 %)	0.277

*ME* McKeown esophagectomy, *TE* thoracolaparoscopic esophagectomy, *T* tumor, *Tis* tumor in situ, *N* node, *M* metastasis, *LN* lymph node, *mD* medium diameter, *G* grade

*p < 0.05; ^*#*^ *p* < 0.0001

Data collected included demographics, overall and thoracic operative times, blood loss during operation and postoperative day (POD) 1, blood transfusion, intensive care unit stay, in-hospital stay, pre- and postoperative tumor node metastasis (TNM) [12] stage analysis, and mortality. Postoperative complications were graded according to the Clavien–Dindo classification [13]. Clavien–Dindo grades I and II represent minor complications, whereas grades III and IV represent major complications [13]. An anastomotic leak (AL) was confirmed by radiology (CT scan or iodine oil contrast esophagography under DSA), endoscopy, or during surgical exploration. The body temperature, heart rate, respiratory rate, white blood cell, C-reactive protein, and incision pain on pre- and postoperative day, POD 1, 3, and 5, were recorded. Numerical rating scales (NRSs) used to assess pain intensity [14]. Patients were routinely followed up for 5 years postoperation according to the following protocol: 1 monthly for 6 months, 3 monthly for 1 year, 6 monthly for 2 years, and yearly thereafter. Recurrence of cancer during follow-up included the first site (anastomosis site), distant organs, local lymph nodes, and distant lymph nodes recurrence.

### Surgical techniques

In ME group, the procedure was the same as described by Siegel et al. [15], in brief, which include thoracic esophageal mobilization through the right fifth or sixth thoracotomy; thoracic lymph node dissection; ligation of thoracic duct (not regularly performed in our department if damaged); abdominal exploration; stomach mobilization; abdominal lymph node dissection; used of nose-duodenal nutritional tube instead of feeding jejunostomy; left cervical incision for anastomosis.

In thoracolaparoscopic esophagectomy (TE) group, at the first stage, all patients were kept in left lateral position and leaning forward. This position obtained better exposure of the posterior mediastinal tissue well by pushing the right lung forward. General anesthesia was performed with single-lumen intubation, which allowed moving trachea for better exposure of the lymph nodes along the right and left recurrent pharyngeal nerve.

Four ports were made in the right chest wall after single ventilation (Fig. 1a): one incision 12 mm in length in the seventh intercostal axillary midline as the observation port for thoracoscope (Fig. 1a(1#); artificial pneumothorax was performed, 8 mmHg); one operation ports about 3 mm in length in the third intercostal anterior axillary (Fig. 1a(2#)); the other two operation ports were made eighth intercostal scapular line (Fig. 1a(4#)) and fifth intercostal between posterior axillary line and scapular line (Fig. 1a(3#)).

After confirming resectable of esophageal cancer, ultrasonic knife and electrocantery were used to cut the low pulmonary ligament and mediastinal pleura on the surface of the esophagus. Arch of azygos vein was dissected and cut after the two ends were clamped by Hemolok. The esophagus was dissected completely from top to bottom. All lymph nodes in the operative field were moved, including lymph nodes of recurrent laryngeal nerve chains (Fig. 1d), paraesophageal, paratracheal, and subcarinal (Fig. 1e). The right chest was closed after a 28 F chest drain was placed through the port 1# and a negative pressure ball place through port 4#. Then, the patient was changed to supine position with the head moving right side to expose the left neck. One 12-mm incision was made under navel for observation port (Fig. 1b(1#); artificial pneumoperitoneum was performed, 12 mmHg); four 5-mm operative ports under subxiphoid (Fig. 1b(3#) enlarged later), right midclavicular line subcostal (Fig. 1b(4#)), left anterior axillary line (Fig. 1b(2#)), and parasternal line 2 cm above navel (Fig. 1b(5#)) were made. Ultrasonic scalpel was used to dissect gastric greater and lesser curvature; the left gastric artery and vein were cut off with two ends clamped by Hemolok. The 3# port under subxiphoid was lengthened to 5 cm, and a 5-cm pipe type gastric was tailored by 75-mm stapler (America Johnson company) from this port. Incision over the front of left sternocleidomastoid muscle was performed and cervical esophagus was isolated. A circular stapler was used to complete cervical esophagogastrostomy.

The postoperative adjuvant chemotherapy was recommended for the patients with advance tumor stage (more than IIB stage). Chemotherapy regimen included Taxol 135 mg/m^2^ on day 1 and cis-platinum 75 mg/m^2^ on day 1. At least four chemotherapy cycles and 21 days as one cycle was recommended. Postoperative radiotherapy is performed when the surgical margin is positive and when there were certain risk factors, such as depth of tumor invasion, lymphovascular invasion, and paratracheal lymph node involvement.

### Statistical analysis

Mann–Whitney *U* and Pearson’s *χ*^2^ tests were performed to test the difference between two groups. The statistical analysis was completed in SPSS® version 19 (SPSS, Inc., Chicago, IL, USA), with *p <* 0.05 indicating a statistically significant difference. Overall and disease-free survival were analyzed by the Kaplan–Meier method calculated from the date of operation until the date of death or date of recurrence, respectively.

## Results

### Demographic parameters

The demographics and operative data of the two groups were described in Tables 2 and 3. The TE and ME groups were matched for age, sex, personal hobbies (alcohol and smoke), preoperative pulmonary function, blood gas analysis, American society of Anesthesiologist score, comorbidities, and blood routine test, except the BMI. The BMI in TE group was significantly lower than that in ME group.

**Table 2:** Patients demographies

	ME group (*n* = 62)	TE group (*n* = 51)	*p*
Age	65.9 ± 6.5	64.5 ± 5.8	0.234
Gender (male/female)	42/20	36/15	0.745
Body mass index (BMI)	25.3 ± 4.6	22.5 ± 5.3	0.003*
Blood gas analysis			
PO2	81.6 ± 7.6	82.6 ± 8.3	0.506
1PCO2	36.4 ± 7.2	35.8 ± 8.6	0.687
Pulmonary function			
Vital capacity %	88.7 ± 13.6	86.9 ± 14.7	0.501
FEV1 %	79.4 ± 4.2	80.8 ± 5.3	0.120
Blood routine test			
White blood cell (10^2^/mm^3^)	57.5 ± 10.8	60.5 ± 11.4	0.155
Neutrophil (%)	71.5 ± 7.8	72.6 ± 8.5	0.475
Hemoglobin (g/L)	121.5 ± 19.4	119.4 ± 22.7	0.597
Albumin (g/L)	34.5 ± 4.3	33.9 ± 4.6	0.476
History of smoke	35 (56.5 %)	26 (51.0 %)	0.561
History of ethanol	23 (37.1 %)	12 (23.5 %)	0.121
Prior gastric or esophageal surgery	2 (3.2 %)	0 (0 %)	0.196
Previous chest surgery	1 (1.6 %)	0 (0 %)	0.362
Comorbidities			
Coronary artery disease	5 (8.1 %)	2 (3.9 %)	0.363
Diabetes mellitus	6 (9.7 %)	7 (13.7 %)	0.502
COPD/emphysema	4 (6.5 %)	1 (2.0 %)	0.248
ASA grade			0.793
I	12 (19.4 %)	11 (21.6 %)	
II	40 (64.5 %)	34 (66.7 %)	
III	10 (16.1 %)	6 (11.8 %)	

*ME* McKeown esophagectomy, *TE* thoracolaparoscopic esophagectomy, *PO2* partial pressure of oxygen, *PCO2* partial pressure of carbon dioxide, *FEV1* forced expiratory volume in 1 s, *COPD* chronic obstructive pulmonary disease, *ASA* American Society Of Anesthesiologists

*p < 0.05

**Table 3:** Operative outcomes data

	ME group (*n* = 62)	TE group (*n* = 51)	*p*
Operation time (min)	252.6 ± 25.8	268.5 ± 34.5	0.006*
Thoracic surgery time (min)	61.2 ± 16.8	69.4 ± 23.6	0.034*
Intraoperative Blood loss (ml)	266.5 ± 98.5	211.8 ± 85.7	0.002*
Blood transfusion (%)	16 (25.8 %)	5 (9.8 %)	0.030
Drainage POD1 (ml)	357.6 ± 102.5	218.5 ± 95.9	<0.0001**
Pulmonary complications (total)	11 (17.7 %)	2 (3.9 %)	0.035
Reintubation	3 (4.8 %)	0 (0 %)	0.111
ICU stay (h)	22.1 ± 5.6	15.7 ± 6.5	<0.0001**
In-hospital stay (days)	15.6 ± 6.8	12.5 ± 4.8	0.007*
In-hospital stay mortality (%)	1 (1.6 %)	0 (0 %)	0.362
Hospitalization cost (dollar)	4856 ± 1255	6583 ± 1643	<0.0001*

*ME* McKeown esophagectomy, *TE* thoracolaparoscopic esophagectomy, *POD1* first day postoperative day, *ICU* intensive care unit

*p < 0.05; **p < 0.0001

### Surgical outcomes

In all patients, digestive tract reconstruction was performed by stapled anastomosis with sleeve gastric pull-up in an orthotopic position. In TE group, one patient converted to open surgery because of adhesion in the right chest cavity. Compared with the ME group, the TE group had a greater overall and thoracic operation time (*p = 0.006* and *p =* 0.034, respectively) and lesser duration of stay in the intensive care unit and in-hospital (*p <* 0.0001 and *p =* 0.007, respectively). The amount of intraoperative blood loss and the incidence of intraoperative blood transfusion were greater in ME group. The drainage of thoracic in ME group was more than that in TE group on POD 1. More pulmonary complications were found in ME group (*p =* 0.035). The expenditure in group TE was higher attributing to the application of endoscopy device. The data is shown in Table 3.

### In-hospital outcomes

There was only one death in ME group (postoperative day 3) from pulmonary embolism because of deep vein thrombosis (DVT) leading to respiratory compromise (confirmed by computer tomography angiography Fig. 1f). The rate of anastomostic leak was not significantly different between the groups (2 vs. 1 patients, *p =* 0.677); all of them were recovered by non-surgery management. Complications according to the Clavien–Dindo classification were reported in 48 patients (42.5 %); the majority (68.7 %) of these was minor complications (grade I or II). A total of 15 patients had a major complication (grade III or IV). There was no statistically significant difference in the rate of major complications between two groups (10 vs. 5, *p =* 0.324). The data is shown in Table 4.

**Table 4:** Postoperative complications of thoracolaparoscopic esophagectomy (TE) compared with open Mckeown esophagectomy (ME)

		Complications	ME	TE	*p*
Clavien–Dindo grade					
Minor	1	Urinary retention	2	1	0.677
		Hypokalemia	2	3	0.400
	2	Atrial fibrillation	8	1	0.033*
		Pulmonary complications	5	1	0.150
		Wound infection	1	0	0.362
		Recurrent nerve injury	4	1	0.248
		Chyle leak	3	1	0.410
Major	3	Pulmonary complications	3	1	0.410
		Chyle leak	2	1	0.677
		Anastomotic leak	1	1	0.889
		Myocardial infarction	0	1	0.268
	4	Pulmonary complications ARDS	2	0	0.196
		Necrosis of gastric tube	0	1	0.268
		Anastomotic leak	1	0	0.362
	5 (death)	DVT/pulmonary embolism	1	0	0.362

*ME* McKeown esophagectomy, *TE* thoracolaparoscopic esophagectomy, *DVT* deep vein thrombosis

*p < 0.05

**Fig. 2:**
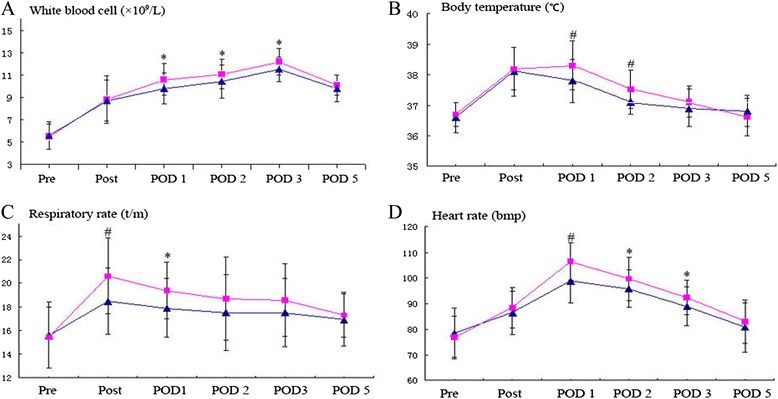
Square (*black square*) represents the ME group; triangle (*black triangle*) represents the TE group. *p < 0.05; ^#^ *p* < 0.0001*.* **a** The number of white blood cell was compared between two groups in pre and 5 days after operation: the number of WBC was significantly less on POD 1, 2, and 3 in TE group. **b** The body temperature was compared between two groups: the temperature was lower on POD 1 and 2 in TE group. **c** The respiratory rate was compared between two groups: the RR was less on POD 0 and 1 in TE group. **d** The heart rate was compared between two groups: the HR was faster on POD 1, 2, and 3 in ME group

**Fig. 3:**
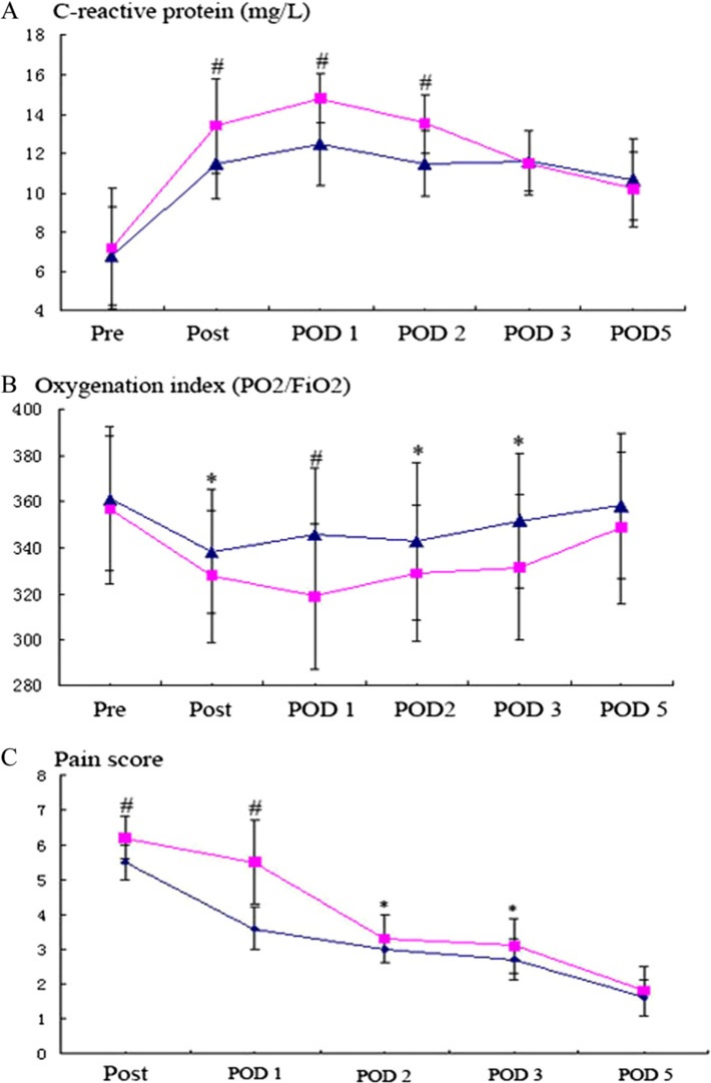
Square (*black square*) represents the ME group; triangle (*black triangle*) represents the TE group. *p < 0.05; ^#^ *p* < 0.0001*.* **a** The C-reactive protein was compared between two groups: the CRP level in blood was significantly lower on POD 0, 1, and 2 in TE group. **b** The oxygenation index was compared between two groups: the OI was better on POD 0, 1, 2, and 3 in TE group. **c** The degree incision pain was compared between two groups: the pain was less on POD 0, 1, 2, and 3 in TE group

The inflammatory response was lighter in TE group than that in ME group. Patients in ME group have significantly higher body temperature on POD 1 and 2, faster heart rate on POD 1, 2, and 3, more white blood cell on POD 1, 2, and 3, faster respiratory rate on POD 0 and 1, higher blood level of C-reactive protein (CRP) on POD 0, 1, and 2, worse oxygenation index (OI) on POD 0, 1, 2, and 3, and more obvious incision pain on POD 0, 1, 2, and 3. The data is shown on Table 5 and Figs. 2 and 3.

**Table 5:** Postoperative inflammation, oxygenation index, and pain score compared between two groups

	ME group (*n* = 62)	TE group (*n* = 51)	*p*
Body temperature (°C)			
Pre	36.7 ± 0.4	36.6 ± 0.5	0.240
Post	38.2 ± 0.7	38.1 ± 0.8	0.480
POD 1	38.3 ± 0.8	37.8 ± 0.7	0.0007**
POD 2	37.5 ± 0.6	37.1 ± 0.4	0.0001**
POD 3	37.1 ± 0.5	36.9 ± 0.6	0.056
POD 5	36.6 ± 0.6	36.8 ± 0.5	0.060
Heart rate (bpm)			
Pre	76.8 ± 8.4	78.6 ± 9.6	0.290
Post	88.4 ± 7.8	86.5 ± 8.4	0.216
POD 1	106.4 ± 7.4	98.7 ± 8.4	<0.0001^#^
POD 2	99.7 ± 8.5	95.8 ± 7.5	0.012*
POD 3	92.4 ± 6.8	88.9 ± 7.6	0.011*
POD 5	82.9 ± 8.4	80.7 ± 9.5	0.194
White blood cell (×10^9^/L)			
Pre	5.5 ± 1.1	5.6 ± 1.2	0.645
Post	8.8 ± 2.1	8.7 ± 1.8	0.789
POD 1	10.6 ± 1.4	9.8 ± 1.4	0.003*
POD 2	11.1 ± 1.3	10.4 ± 1.5	0.009*
POD 3	12.2 ± 1.2	11.5 ± 1.1	0.002*
POD 5	10.1 ± 0.9	9.8 ± 1.2	0.132
Respiratory rate (t/m)			
Pre	15.4 ± 2.6	15.6 ± 2.8	0.695
Post	20.6 ± 3.2	18.5 ± 2.8	0.0004**
POD 1	19.4 ± 2.4	17.9 ± 2.5	0.0016*
POD 2	18.7 ± 3.5	17.5 ± 3.2	0.062
POD 3	18.6 ± 3.1	17.5 ± 2.9	0.056
POD 5	17.3 ± 1.9	16.9 ± 2.2	0.302
C-reactive protein (mg/L)			
Pre	7.2 ± 3.1	6.8 ± 2.5	0.459
Post	13.4 ± 2.4	11.5 ± 1.8	<0.0001^#^
POD 1	14.8 ± 1.3	12.5 ± 2.1	<0.0001^#^
POD 2	13.5 ± 1.5	11.5 ± 1.7	<0.0001^#^
POD 3	11.5 ± 1.6	11.6 ± 1.5	0.735
POD 5	10.2 ± 1.9	10.7 ± 2.1	0.187
Oxygenation index (PO2/FiO2)			
Pre	356.5 ± 32.2	361.2 ± 31.5	0.437
Post	327.6 ± 28.6	338.4 ± 26.8	0.042*
POD 1	318.6 ± 31.6	345.6 ± 28.9	<0.0001^#^
POD 2	328.8 ± 29.6	342.6 ± 34.3	0.023*
POD 3	331.5 ± 31.7	351.6 ± 29.5	0.001*
POD 5	348.8 ± 32.8	358.4 ± 31.5	0.118
Pain score			
Post	6.2 ± 0.5	5.5 ± 0.6	<0.0001^#^
POD 1	5.5 ± 0.6	3.6 ± 1.2	<0.0001^#^
POD 2	3.3 ± 0.4	3.0 ± 0.7	0.005*
POD 3	3.1 ± 0.6	2.7 ± 0.8	0.003*
POD 5	1.8 ± 0.5	1.6 ± 0.7	0.079

*ME* McKeown esophagectomy, *TE* thoracolaparoscopic esophagectomy, *pre* preoperative, *Post* postoperative, *POD* postoperative day, *bpm* beats per minute, *t/m* times per minute, *PO2* partial pressure of oxygen, *FiO2* fraction of inspired oxygen

*p < 0.05; **p <0.001

^*#*^ *p* <0.0001

### Pre- and postoperative pathologic parameters

The TNM stage difference between two groups is shown in Table 1. In preoperative tumor staging, T2 (27.4 %) and T3 (38 %) were detected in ME group, while T1 (29.4 %) and T2 (49 %) were detected in TE group (*p <* 0.0001), which was similar in postoperative tumor staging (*p =* 0.001). However, both in ME and TE group, there was no significant difference between pre- and postoperative T staging (*p >* 0.05), respectively. Preoperatively, more lymph nodes were found in ME group from the CT scan, but more lymph nodes were removed in TE group; there was no significant difference on mean positive nodes between two groups. There was no difference on the tumor morphology and differentiation degree between two groups. There was no significant difference on the postoperative adjuvant therapy between two groups (Table 1).

### Survival and medium-term outcomes

**Fig. 4:**
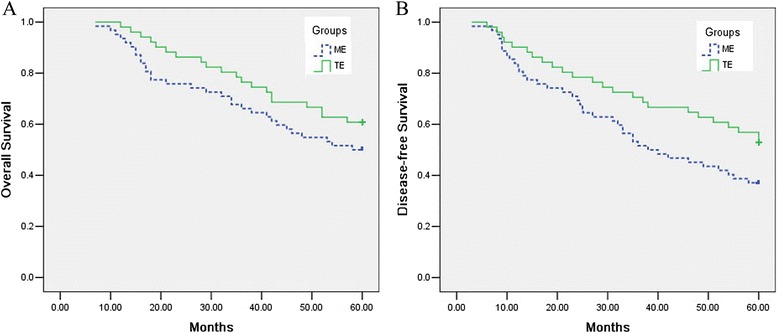
*Solid line* (*—*) represents the TE group; *dotted line* (*---*) represents the ME group. **a** The overall survival rate was compared between two groups (log–rank test: *p* = 0.193). **b** The disease-free survival rate was compared between two groups (log–rank test: *p* = 0.065)

The median follow-up was 48 months (range 30–60 months, ME 52 months vs. TE 44 months). No significant difference was found between two groups on overall survival (Fig. 4a, log–rank test: *p =* 0.193) or disease-free survival (Fig. 4b, log–rank test: *p =* 0.065). A total of 52 patients (46 %) died during follow-up (ME 32 vs. TE 20). Disease recurrence was observed in 61 patients (54 %) in 5 years, and there was no statistical difference between two groups.

## Discussion

McKeown esophagectomy was the main surgical approach for the middle esophageal cancer nowadays in China. ME had the advantage of reducing local recurrence and easier to manage if anastomosis leak occur in the neck; however, the trauma caused by ME was great and the postoperative complications happens frequently [6]. With the development of the laparoscopy and thoracoscopy technology, combined thoracolaparoscopic esophagectomy for esophageal cancer was increasingly adopted by thoracic surgeons. Theoretically, TE could offer the potential advantages of rapid recovery, alleviating of incision pain, and restore to normal function promptly; however, the postoperative outcomes were controversial [16]. The data of 51 patients undergoing TE in our department were collected and analyzed. The postoperative outcomes were compared carefully between two groups, which especially included the postoperative complications, Systemic Inflammatory Response Syndrome (SIRS), and incision pain.

The surgery time of TE was significantly longer than that of the ME group both in overall and thoracic operation. This result was consistent with several previous studies [17, 18]. But Sundaram et al. [19] reported that the operative time of TE was significantly shorter than that of conventional transthoracic esophagectomy. TE is a technically advanced surgical procedure which needs a relatively longer learning curve. It has been reported that a minimum of 17 cases were necessary to acquire TE skills, and more than 35 cases were needed to achieve an outcomes difference [20, 21]. Most of the patients in TE group were in the beginning of the learning curve. So, the surgical time was determined by surgeon’s proficiency. Less blood loss and transfusion were observed in TE group which was also found in series researches [17–19]. Good visualization of the surgical field during TE may ensure hemostasis and thus contribute to reducing the blood loss.

Whether TE could achieve equivalent or superior oncologic outcomes with ME, two aspects included en bloc tumor resection and field of lymphadenectomy. In this study, no positive margin of esophagus was detected in both groups, which indicated that the tumor can be removed completely by both two approaches. However, the number of retrieved lymph nodes in TE group was significantly greater than that in group ME (17.8 ± 3.6 vs. 15.3 ± 3.2, *p =* 0.0002). The view of the surgical field under TE was magnified which contribute the more retrieved lymph nodes. Positive lymph node along the right recurrent laryngeal nerve (RLN) was found in 15 patients (11 in TE group and 4 in ME group); this may attribute to the more lymph nodes retrieved along recurrent laryngeal nerve. Under the thoracoscopy, the recurrent laryngeal nerve can be dissected and protected carefully. Four patients in ME and one patient in TE have RLN palsy. The only one patient in TE group with RLN palsy attributed to the RLN enwrapped by cancer, and the RLN was injured by the high thermal energy of ultrasonic scalpel. All of the nerve injury was temporary, and the laryngeal function gradually returned to normal in few weeks. However, not all the surgical team encountered the same condition. Schoppmann and Ben-David [22, 23] reported the converse results.

In this study, more patients in ME group was in advanced TNM stage. This is an inherent limitation of the retrospective study. The patients undergoing combined thoracoscopic and laparoscopic surgery were selected strictly. Patients in both groups received barium swallow X-ray examination, esophagogastroduodenoscopy with biopsy, and chest and abdominal computed tomography to estimate the preoperative tumor TNM stage. In our department, the selected patients for TE were usually without serious comorbidities, and the esophageal tumor stage (T stage) of them was much earlier when compared with ME group (*p* < 0.001).

Preoperative tumor stage (T stage) was mainly appraised by endoscopic ultrasonography (EUS). EUS can provide information on invasive depth of EC (T stage) and locoregional lymphadenopathy (N stage) [24, 25]; however, recently prospective research revealed that EUS was limited on resectability and the authors suggested that it should not be performed in all patients before surgery [26]. The patients with EC in our department were received EUS examination before surgery. In this cohort study, the preoperative T stage evaluated by EUS was comparable with the postoperative T stage (*p* > 0.05). We thought that EUS was a useful method for the accurate staging of esophageal cancer before surgery.

Postoperative AF can result in hemodynamic compromise, thromboembolic phenomena, and anxiety. Other sequelae include prolonged length of stay (LoS) and increased cost. Several studies [27–29] indicated that AF after esophagectomy was associated with postoperative inflammatory response, pulmonary complications, surgical trauma, hypoxia, incision pain, and damage of sympathovagal nerve fibers supplying heart. Previous published papers [27, 28] try to elucidate the relation between AF and anastomotic leak; however, there is no determination of whether AF is caused by leak or, conversely, leak is caused by AF with low cardiac output [30]. In this study, the results did not allow to explicit the relation between them. Three patients suffered with anastomotic leak (occurred on POD 4, 5, and 7, respectively); two of them have new onset AF on POD2 and last for 2 and 4 days, respectively. The cervical gastroesophageal anastomosis was performed in all patients in this study, even if the leak happened; the digestive juice can be drained from the cervical incision without effect on thoracic cavity.

However, the relation between AF and pulmonary complication was strong. All nine patients with AF had pulmonary complications in varying degrees. The results were consistent with previously published series [27, 28, 31, 10]. More patients with AF and pulmonary complication was found in ME group when compared with TE group (8 vs. 1, *p* = 0.033; 11 vs. 2, *p* = 0.035). The pulmonary complications were mainly pneumonia and hypoxia (oxygenation index lower than 300). Postoperative pneumonia happened frequently after open esophagectomy. These kinds of patients usually present with cough, expectoration, fever, wheezy phlegm by auscultation, and lower SpO2. Re-intubation was performed on three patients with pneumonia in ME group who could not excrete the phlegm. Minimal invasive esophagectomy offers the advantage of alleviating the incision pain and promoting earlier expectoration.

Open esophagectomy can cause obvious inflammatory response, leading to the postoperative complication, minimal invasive esophagectomy can attenuate SIRS [32, 33]. SIRS consist of four simple and clinical common used indicators, which were used to evaluate the severity of surgical stress [32, 34]. In this study, we found that the incidence of SIRS in TE group was decreased. The more trauma caused by operation and the more severe inflammatory response happened. Operative trauma can activate the inflammatory cell following releasing some pro-inflammatory cytokines which lead to a series postoperative complications. One of effective methods to attenuate SIRS was to decrease operative trauma. Minimal invasive esophagectomy such as TE has the advantage of minimal injury, less blood loss, less blood transfusion, less incision pain, which alleviate the surgical stress.

Postoperative incision pain is a common phenomenon in both surgical approaches. In this study, the patients in ME group suffered greater incision pain until POD5. This result was consistent with the report by Borro et al. [35], which was that the incision pain caused by surgery just lasted for 4 days. The incision pain was closed associated with postoperative complications. Many patients in ME group complained that they dared not coughing because of incision pain, which contributed to the pneumonia, atelectasis. Incision pain could also induce tachycardia, which was also a cause of AF.

### Limitation of the study

The main limitation was the prospective study, and case capacity was not large enough. As to the technique of thoracolaparoscopic esophagectomy was performed since April 2010, at the beginning, the patients with lower BMI and earlier tumor stage were selected in minimal invasive group. All these led to the bias. Nowadays, more patients with great BMI and advanced stage were treated by thoracolaparoscopic esophagectomy. Furtherly, new prospective randomized controlled trials are needed to validate the results.

## Conclusions

In this cohort study, although it is lack of randomness in this research, SIRS of the patients in TE group was lighter, and TE approach could alleviate the postoperative incision pain, both contribute to better oxygenation index after operation and lower postoperative pulmonary complications. AF happened less in TE group probably because of lighter inflammatory response, less pulmonary complications, less incision pain, and better OI. The survival was similar between two groups. The efficacy and safety of TE were supported by these selected patients.
